# Decay of Teeth

**Published:** 1883-01

**Authors:** 


DR. FRANZIUS finds that the tooth most often affected by decay is the third molar, such cases forming one-half of the total number. The teeth begin to decay in a certain successive order, the lower third molar being first attacked, then the upper, then the lower fourth molar, and so on, the incisors and the canine teeth of the lower jaw being the last reached. The upper teeth are more durable than the lower, in the proportion of three to two. The right teeth show a greater vitality than the left. The durability of teeth is less in light persons than in dark, and less in tall than in short persons. These results were obtained by an examination of 650 Russian soldiers, of whom 258 had unsound teeth.

ANOTHER pet idea in natural history must go. The kangaroo does not stand upon a tripod, nor make his vast leaps by spurning the ground with his tail. To prove this, a band of kangaroos obligingly crossed a space of wet sand in the presence of Mr. Nicolls, an Australian naturalist, and left exact impressions of their tracks. Only one accidental mark showed an impress of the tail upon the sand, though the great hind feet left deep tracks, some of which showed 20 feet as the average length of the leaps, which is believed to be often exceeded. Mr. Nicolls believes, however, that the massive tail performs an important part in balancing the body and bringing it to the point of departure for each successive stride, and, so far as can be observed during the excitement of the chase, appears, by being swung to one side, to help the animal in making those sharp doubles which confound the best dogs.

